# Study on the mechanism of inhibiting patulin production by fengycin

**DOI:** 10.1515/biol-2022-0041

**Published:** 2022-04-19

**Authors:** Ruimin Fu, Wei Tang, Hong Zhang, Yulian Zhang, Ding Wang, Wuling Chen

**Affiliations:** College of Health Management, Henan Finance University, Zhengzhou, Henan, China; College of Life Science, Shaanxi Normal University, Xi’an, Shaanxi, China

**Keywords:** *Penicillium expansum*, patulin, fengycin, 6-MSAS, IDH

## Abstract

*Penicillium expansum* is the main cause of apple rot. Besides, it can also produce mycotoxin patulin (PAT). Therefore, the search for substances that can inhibit the activity and toxigenicity of *P. expansum* has become a hot research topic. This study investigates the inhibitory effects of fengycin on patulin production in *P. expansum. P. expansum* was cultured under different environments with different concentrations of fengycin. The patulin content produced per unit weight of *P. expansum* mycelium was detected and determined by high pressure liquid chromatography (HPLC). Synergy brands (SYBR) GreenI Real-time PCR was used to detect the expression levels of 6-methylsalicylic acid synthase (6-MSAS) and isoepoxydon dehydrogenase (IDH), which were the key genes of producing patulin of *P. expansum* mycelium, in the conditions treated by fengycin and untreated. After fengycin treatments, not only the patulin content in every unit weight of *P. expansum* mycelium but also the expression level of 6-MSAS decreased significantly. The expression level of 6-MSAS of treatment was 0.11 folds of control. However, the expression level of IDH treated by fengycin decreased slightly. Fengycin could inhibit the *P. expansum* from producing patulin by downregulating the expression of key synthetic genes 6-MSAS.

## 1 Introduction

*Penicillium expansum* is the main pathogenic fungus that causes post-harvest diseases in apples. It causes the fruits to decompose and produce the mycotoxin patulin (PAT) [1,2]. This study has indicated that PAT’s carbon skeleton is constructed by polyketide synthase. In the previous study, we found that fengycin can inhibit the growth of *P. expansum in vitro* and in apple fruits [3] and reduce the accumulation of toxins secreted by pathogens [4]. A strain, BA-16-8, which could inhibit the growth of *P. expansum,* was isolated, screened, and identified as *Bacillus amyloliquefaciens* in the previous study.

We speculate that *B. amyloliquefaciens* either inhibits the production of Patulin (PAT) by *P. expansum* or degrades PAT [5].

Real-time quantitative polymerase chain reaction (RTQ-PCR) is a technique to quantify the initial template amount in the PCR product. The PCR product in each cycle of the reaction is monitored in real-time to add a fluorescent group to the PCR reaction system and detect the accumulation of fluorescence signal intensity by a specific instrument. The technology is mainly carried out by the intensity of the fluorescent substance. The common fluorescence quantification methods include synergy brands (SYBR) Green intercalating dyes and the probe method [6,7]. SYBR Green intercalating dyes were conducted by intercalating the SYBR Green into the minor groove of double-stranded DNA, for which green fluorescence can be emitted after combining, and the amount of DNA template can be quantitatively synthesized based on the intensity of fluorescence signal by the fluorescence q-PCR instrument [8,9]. Real-time fluorescent q-PCR has advantages such as high sensitivity, strong specificity, convenient use, high throughput, no ethidium bromide contamination, and others [10], which has been widely used in pathogen detection and gene expression differential analysis [11].

The genes that control the production of PAT in the cells of *P. expansum* are clustered in the genome, among which 6-methylsalicylic acid (6-MSAS) and isoepoxydon dehydrogenase (IDH) are shown as the key genes [12]. In order to explore the effect of fengycin as a metabolite of *B. amyloliquefaciens* on the expression of PAT in *P. expansum*, this study intends to detect the PAT content produced per unit weight of *P. expansum* mycelium through HPLC. Based on different concentrations of fengycin, the expression of target genes 6-MSAS and IDH is analyzed by real-time fluorescent q-PCR technology, which verifies the inhibitory effect of fengycin on PAT [13,14,15]. The study aims to investigate the inhibitory effect of fengycin on patulin production in *P. expansum.*

## 2 Materials and methods

### 2.1 Materials

Fengycin was separated and purified in our laboratory, referred to in the literature [2,16]. The PAT standard substances were purchased from sigma. Trizol was purchased from Invitrogen, the United States. RNA extraction kit and reverse transcription kit were purchased from Promega, the United States. LightCycler 2.0 real-time quantitative PCR instrument (Roche) and SYBR Green fluorescent dye kit (Takara) was applied. Pre-stained molecular weight Marker (Fermentas), ethanol, isopropanol and diethypyrocarbonate, and other reagents were domestically analytical reagents. Potato Dextrose Agar and Potato Dextrose Broth (PDB) were formulated explicitly in reference to the literature [17].

*P. expansum* was transferred to a PDB medium and incubated at 30°C and 180 rpm for 48 h. The obtained spore suspension was used to detect toxin production and RNA extraction of *P. expansum* per unit mass of mycelium.

### 2.2 Detection of PAT produced by *P. expansum* per unit mass of mycelium

Fengycin was collected and purified in reference to the literature [2,16]. Taking mycelium without fengycin treatment as control, the PAT produced by *P. expansum* per unit mass of mycelium was weighted and calculated, as referred to in the literature [17].

Referred to the literature [18], the detection of PAT was separated and purified by a C18 reverse-phase column. The HPLC analysis system used in the experiment was the Agilent HPLC 1200 series. The chromatographic column was ZORBAX Eclipse XDB-C18 Analytical Column (5 μm, 4.6 nm × 250 mm). The detection wavelength was 276 nm with a column temperature of 35°C. The proportion of the mobile phase water A (acetonitrile contained with 0.1% trifluoroacetylacetone (TFA)) to the mobile phase water B (water contained with 0.1% TFA) was 1:9, on the flow rate as 1 mL/min. The injection volume was 20 μL. The standard and the sample were injected in an equal amount of 20 μL for quantification by the external standard method.

### 2.3 RNA extraction from *P. expansum*

Taking the spore suspension without fengycin as control, the spore suspension of *P. expansum* was added with fengycin to make the final concentration be 50 µg/mL. After 7 days of culture, the mycelium of both the experimental and control group was centrifuged and freeze-dried. Referred to the literature [19], RNA was extracted from the obtained powder using an RNA extraction kit (Promega, USA).

### 2.4 Reverse transcription PCR

As for complementary DNA (cDNA) synthesis, RT-PCR was performed with reference to the literature [20]. The first cDNA strand was synthesized using RNA extracted from *P. expansum* as a template. The reaction processes were kept at 30°C for 10 min, 42°C for 20 min, 99°C for 5 min, and 4°C for 5 min respectively for one cycle. The cDNA was obtained after centrifugation with the reaction system as 10 μL: 2 μL of MgCl_2_ (25 mmol/L), 1 μL of 10 × RT Buffer, 3.75 μL of Rnase-free dH_2_O, 1 μL of dNTP mixture (2.5 mmol/L), 1 μL of Rnase Inhibitor (40 U/μL), 0.5 μL of AMV Reverse Transcriptase XL (200 U/μL), 0.5 μL of Oligo dT-Adaptor Primer (10 μmol/L) and 1 μL of RNA.

### 2.5 SYBR Green I real-time PCR

The method of RNA extraction from *P. expansum* was conducted as referred to in the report [21]. According to the gene sequence of the PAT synthetic gene 6-MSAS and IDH of *P. expansum* in GenBank, the software Primer Selection 3.0 provided by ABI for designing fluorescent q-PCR primers was designed for primer of SYBR Green I real-time PCR. Fungal universal primers were taken as the internal reference gene 18S rRNA, which was optimized for use. The internal control was designed to avoid errors caused by incorrect RNA quantification. The forward and reverse primer sequence design of each of the above-mentioned genes was shown in Table 1, and the primer synthesis was completed by TaKaRa Company.

Reactions were carried out by using 6.4 μL of Rnase free H_2_O, 1 μL of deoxy-ribonucleoside triphosphate (dNTP) (10 mmol/L), 0.8 μL of forward primer (10 mmol/L), 0.8 μL of reverse primer (10 mmol/L), 10 μL of 2 × SYBR Green I, and 1 μL of the first strand template of cDNA. Real-time PCR was performed with ABI’s Power SYBR Green Master Mix on the applied instrument as Biosystem 7500 Real-time PCR System. Thermal cycling for these data consisted of an initial step at 95°C for 3 min, and 40 cycles of denaturation at 95°C for 40 s plus annealing at 60°C for 40 s. Fluorescence was collected at the annealing stage of each cycle.

**Table 1:** Primer information

Primer	Coding product	Sequence	Annealing temperature (°C)
6-MSAS	6-Methylsalicylic acid synthase	F: CGAAATCGCGGCCAGTGTTGTGR: MGACCATGTTGCCGGCCCAGTATTC	60
IDH	Isoepoxydon dehydrogenase	F: GGNGARGCNATGGTNCATAARTTR: CCAATGYTCNGTCTCNCCCTCCATATG	58
18SrDNA	18SrRNA	F: GCTCTTTTGGGTCTCGTAATTGGR: CGCTATTGGAGCTGGAATTACC	55

### 2.6 Preparation of 18S rRNA standard curve

The control cDNA obtained after reverse transcription was serially diluted, and SYBR Green I RT-PCR was performed on each serial dilution of the internal reference gene 18S rRNA by optimizing conditions. The logarithm of the relative concentration of the relative initial RNA was taken as the abscissa. The relative quantitative standard curve was established with a Ct value as ordinate.

### 2.7 Data processing

After completing the real-time fluorescence q-PCR, the obtained data were analyzed by ABI7500 SDS software (Applied Biosystems, Inc.), and the relative expressions of each gene in different samples were calculated by the 2^−ΔΔCt^ method based on the Ct value of the target gene and the internal reference gene. In this process, ΔΔCt = (CT target gene – CT internal reference) experimental group – (CT target gene-CT internal reference) control group, the data were processed by SAS statistical software. Afterward, the significance test was conducted after logarithmic transformation. The difference shall be considered significant (*) if *P* < 0.05, the difference would be considered a highly significant difference (**) if *P* < 0.01.

## 3 Results and analysis

### 3.1 Impact of Fengycin on PAT

The production of PAT was detected by HPLC and the results showed a retention time of 7.743 min at a wavelength of 276 nm on the corresponding chromatogram. From the peak area control standard sample, we can determine and calculate the amount of PAT, which was determined based on the following equation:

X=A\text{/Am}\times C]

*X – *PAT content of the sample to be tested (mg/L); *A* – PAT area of the sample to be tested (mm^2^); Am – standard PAT area (mm^2^); *C – *PAT concentration of the standard sample (mg/L).

After calculation, we found that under the treatment of fengycin at a concentration of 50 μg/mL, the amount of PAT produced by the mycelium per unit weight of *P. expansum* was significantly lower than the control group, which indicated that the toxin-producing ability of the treated mycelium had decreased. The test result was the same as Figure 2, which showed that although the production time of *P. expansum* was the same as that of the control, under the influence of fengycin, its yield and rate of increase were much lower than the control.

**Figure 1:**
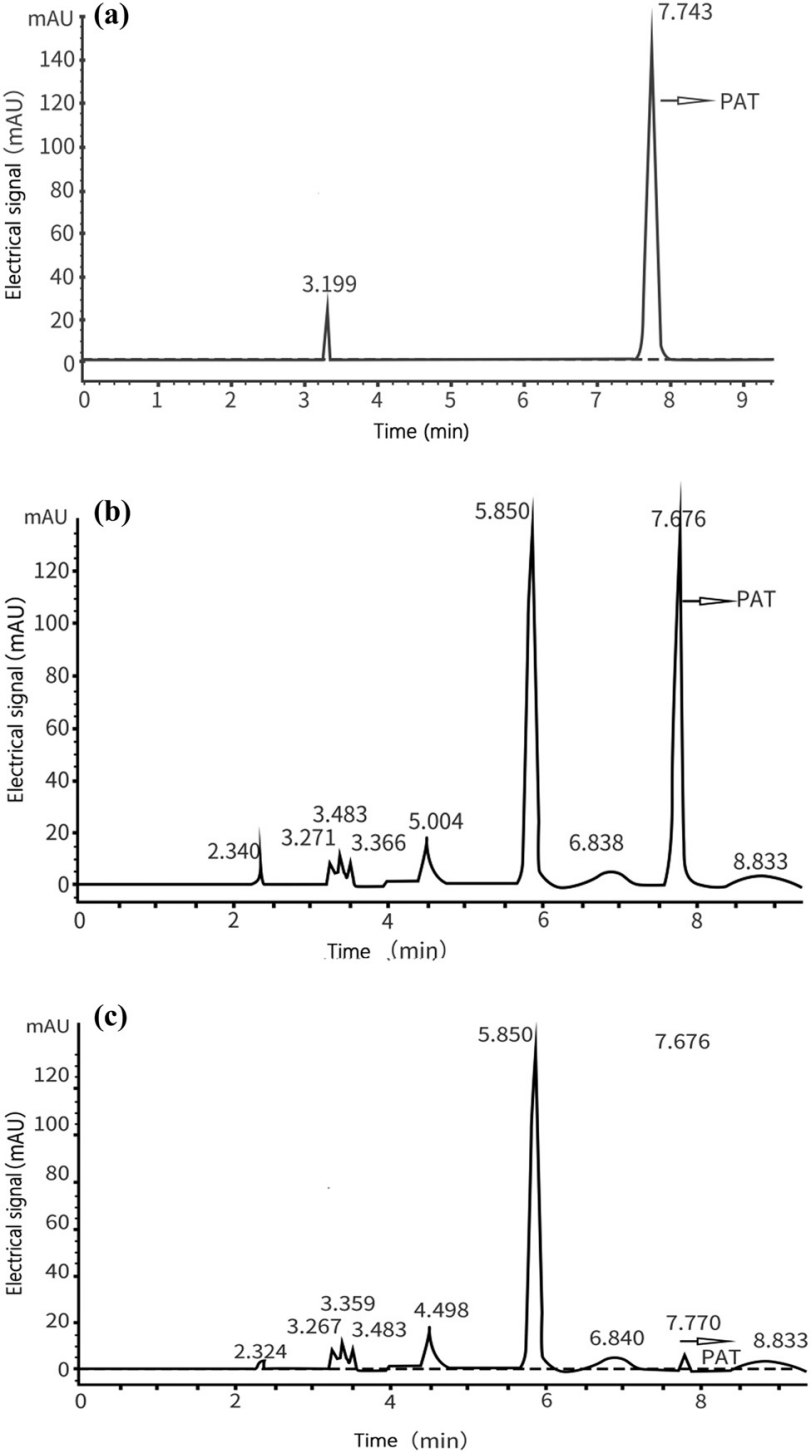
HPLC chromatograms of PAT. (a) A 200-ng sample of standard PAT; (b) PAT extracted from PDB medium culture of control *P. expansum*; (c) PAT extracted from PDB medium culture of control *P. expansum* treated by fengycin.

**Figure 2:**
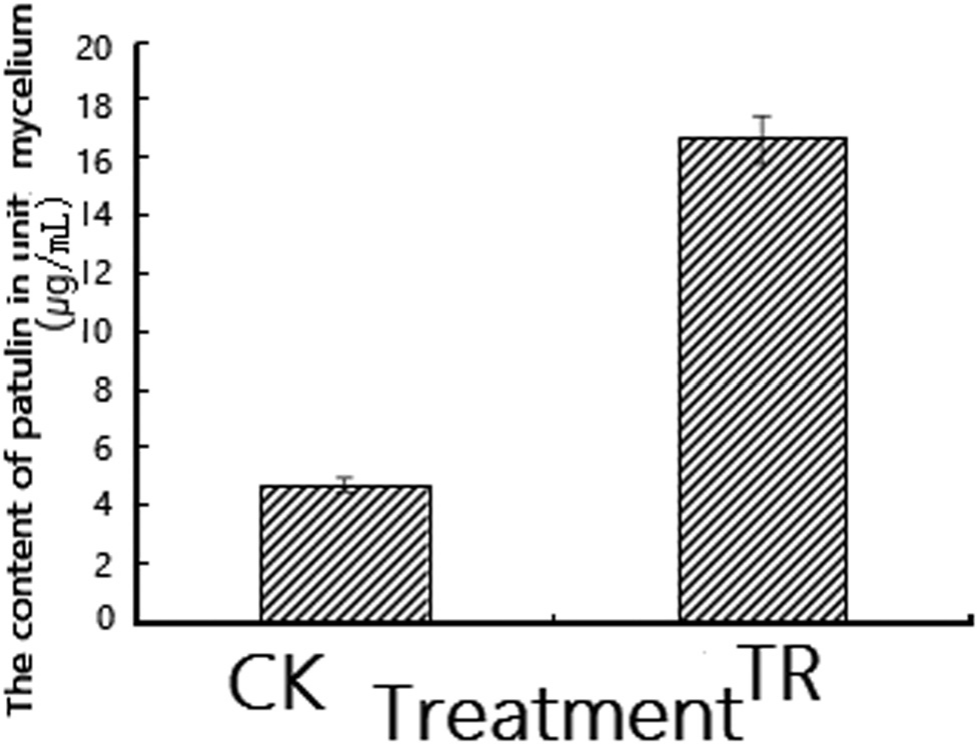
Effect of fengycin on the production of PAT by *P. expansum.* TR: samples treated by fengycin; CK: control samples.

### 3.2 The effect of fengycin on the 6-MSAS and IDH genes’ transcription of *P. expansum*

The total RNA of the control and the experimental groups was detected by agarose gel electrophoresis, shown by the clear bands. The 28S and 18S bands were particularly clear, and the brightness of 28S was twice that of 18S, which indicates that the total RNA mentioned was complete without degradation. After analysis and comparison of A260/A280, the values were recorded as 1.8–2.0, indicating that RNA has good purity that can be used for PCR amplification.

**Figure 3:**
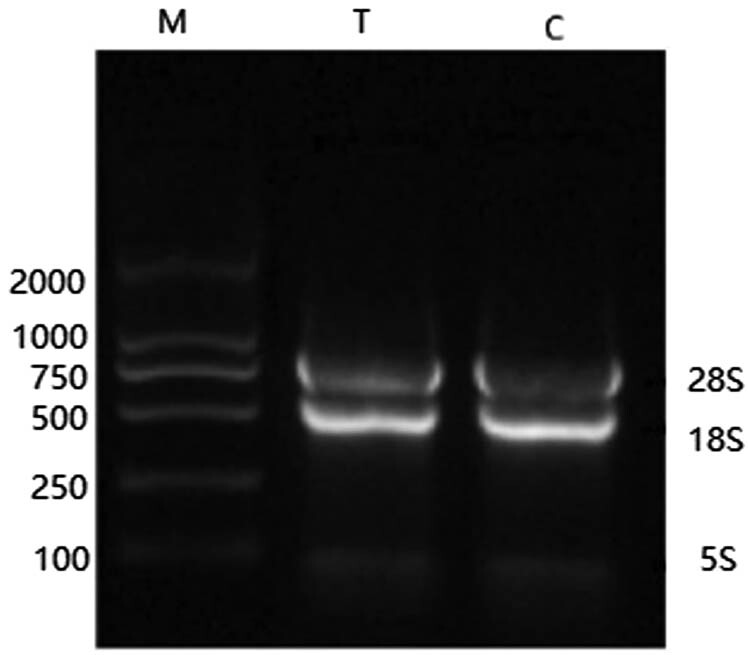
Electrophoresis of total RNA extracted from *P. expansum.* M: marker T: *P. expansum* treated with fengycin C: control samples.

The extracted total RNA was diluted to a 1 µg/mL concentration, and reverse transcription was performed after digestion with DNase. The internal reference gene 18SRNA was amplified by PCR to detect the quality of cDNA obtained by reverse transcription. The gel electrophoresis results of PCR products are shown in the following Figure 3.

**Figure 4:**
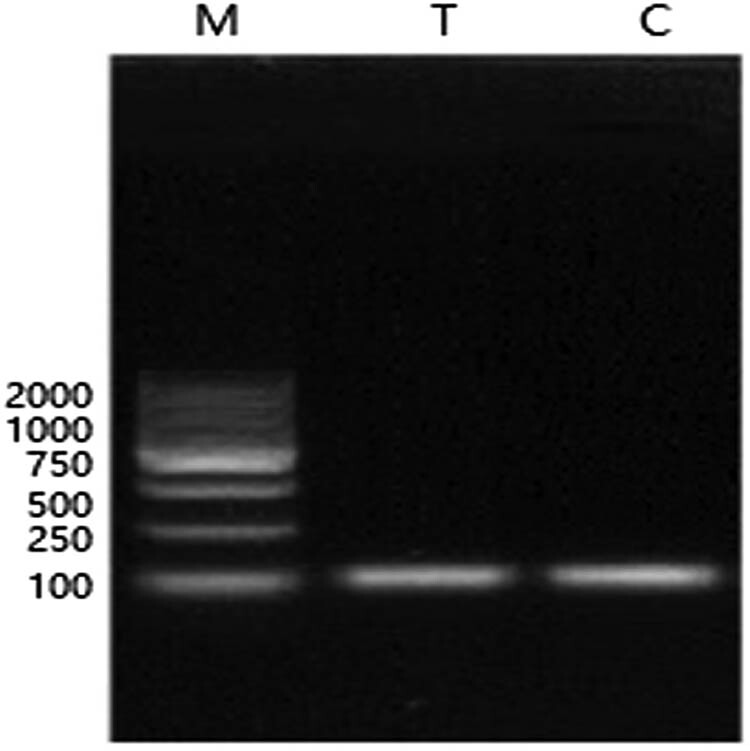
Electrophoresis of total 18srRNA extracted from *P. expansum.* M: marker T: *P. expansum* treated with fengycin C: control samples.

As can be seen in Figure 4, the cDNA of the treatment group and the control group had a bright and single band on the PCR amplification product of internal reference gene 18SRNA, which was the same size as the target band (about 110 bp), with no obvious dimerization shown. There were also no non-specific amplified bands, indicating that the cDNA was obtained by reverse transcription from the total RNA extracted by *P. expans*um without genomic contamination, which can be used as the template for detecting gene expression in the next RT- PCR experiment.

**Figure 5:**
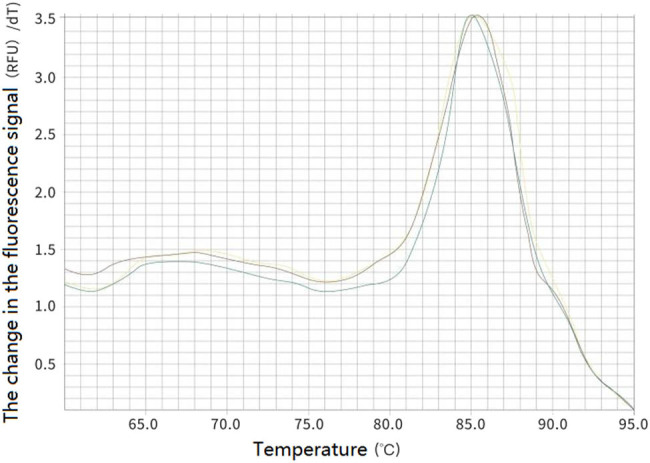
The melt curve of three genes in *P. expansum.*

In order to detect the specificity of the designed primers, real-time fluorescent PCR reactions were performed on the three genes. As shown in Figure 5, the melting curves for the three genes had only a single peak, indicating that the designed primers were specific and can be used to detect gene expression situations.

**Figure 6:**
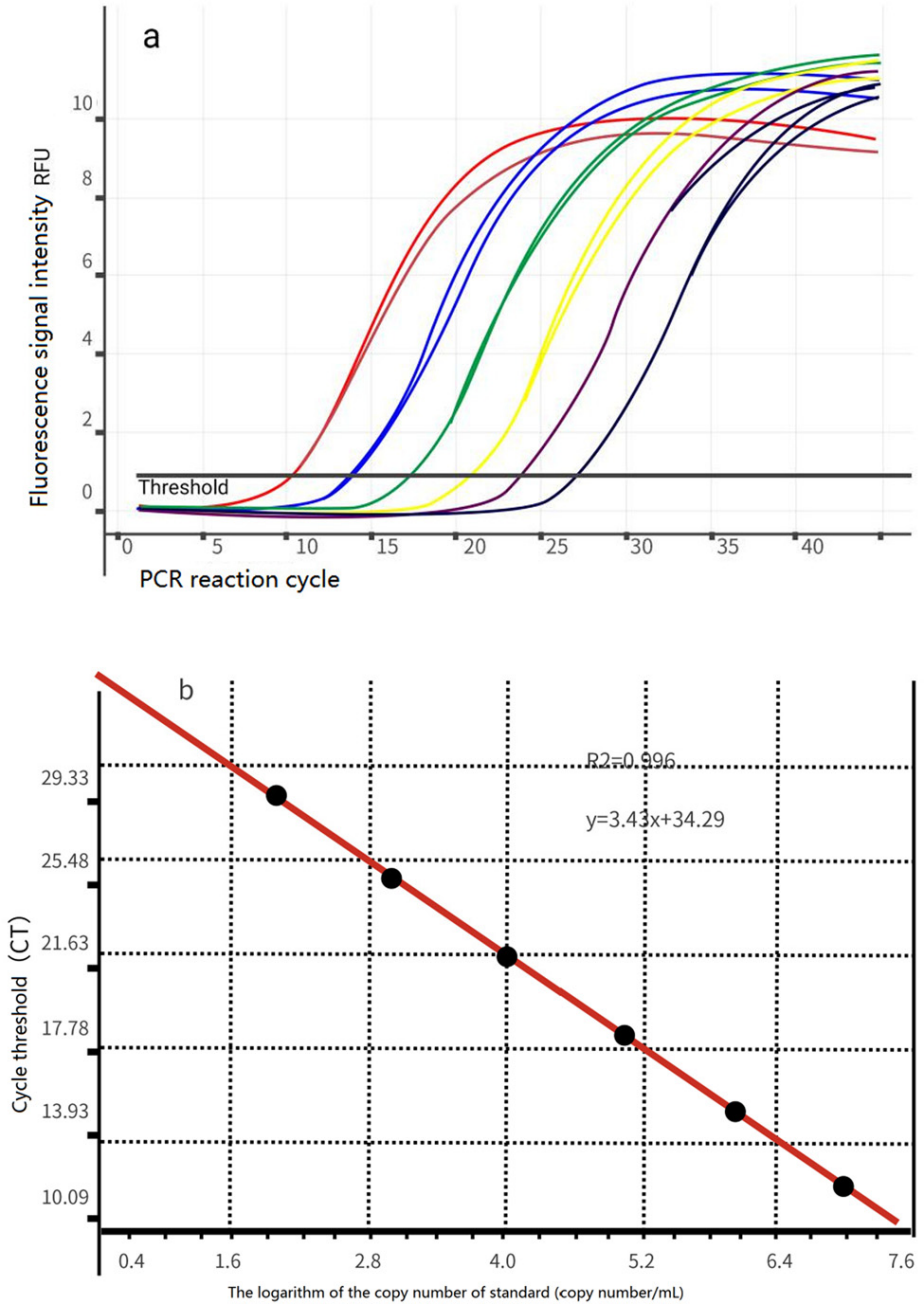
Amplification curve (a) and standard curve (b) of 18S rDNA by RT-PCR.

The internal reference gene 18SRNA was 10-fold diluted and the number of copies measured by ASP-3700 was 1.2 × 10^6^, 1.2 × 10^5^, 1.2 × 10^4^, 1.2 × 10^3^, 1.2 × 10^2^, and 1.2 × 10^1^, and one internal reference gene in each concentration was selected. They were used as a template to perform PCR reactions and optimize the reaction conditions. Finally, the corresponding S-type fluorescence q-PCR amplification curve was obtained, with the logarithm of the standard as the abscissa and Ct as the ordinate to obtain the standard curve of internal reference gene 18SRNA (Figure 6). The results showed the standard curve of internal reference gene *R* ^2^ = 0.996, indicating that the conditions meet the quantitative requirements and the gene expression can be analyzed by 2^−δδCt^ method.

The 2^−δδCt^ method was used to analyze the effect of fengycin on the mRNA expression level of the target gene of *P. expansum*. The samples used in the experiment were the mycelium of *P. expansum* treated with fengycin and the mycelium of *P. expansum* without fengycin.

**Figure 7:**
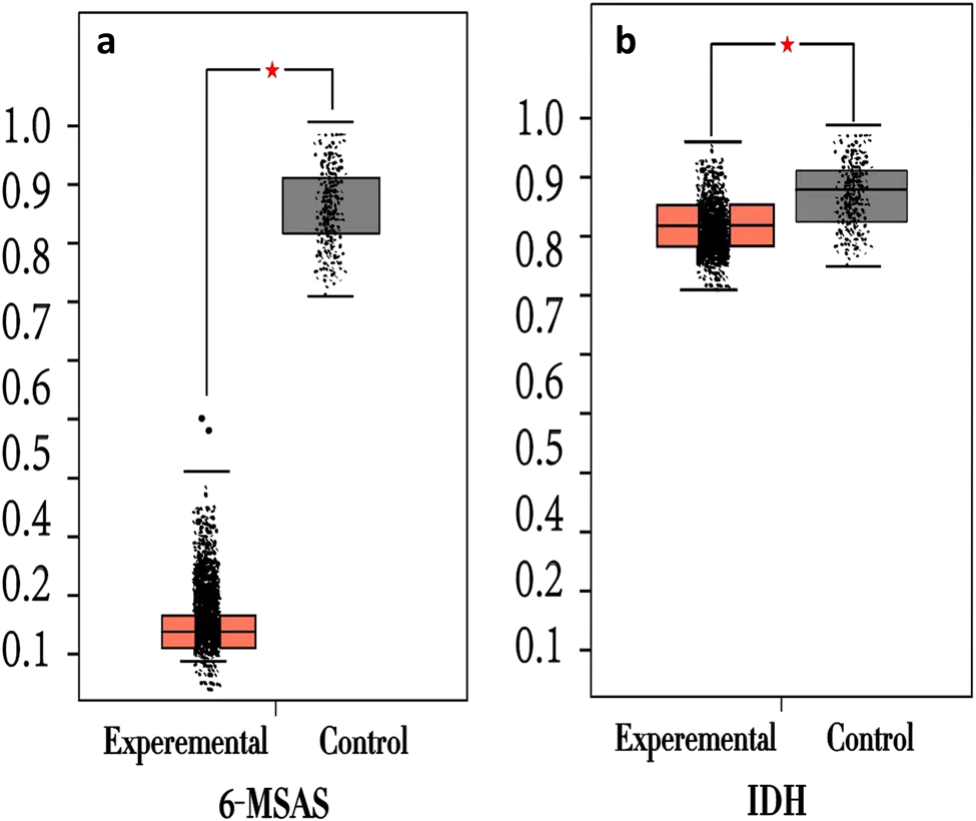
Expression of 6-MSAS (a) and IDH (b) in the fungal mycelium of experimental group and control group.

Quantitative analysis of mRNA expression levels of 6-MSAS and IDH in the samples treated with fengycin was corrected by internal reference 18SrDNA. The mRNA expression levels of 6-MSAS and IDH in *P. expansum* without fengycin treatment were used as a benchmark, and the changes in the mRNA expression levels of 6-MSAS and IDH after the addition of fengycin were calculated and expressed in multiples. The results are shown in Table 2 and Figure 7. It was found that fengycin was able to cause a significant decrease in the expression level of the 6-MSAS gene in *P. expansum* mycelium, which was 0.11 times that in the control group. However, even though IDH gene expression decreased, the difference was not significant compared with the control group.

## 4 Discussion

PAT is a fungal toxin produced by *P. expansum*. Vladimir Ostry et al. [22] provide new information on the potential risk represented by the presence of PAT in grape, confirming that these mycotoxins and *P. expansum* could pose a threat to human health. Not only in the grape but the excessive amount of PAT in apple by-products such as fruit juice caused by infection of *P. expansum* has become a major factor restricting its international development. Therefore, the search for an effective way to restrain *P. expansum* growing activity and toxin-producing ability has become the main task to control the blue decay of post-harvest apples [23]. In a previous study, we found that the cell-free fermentation broth of strain BA-16-8 could significantly reduce patulin accumulation in *P. expansum* mycelium. However, it had no degradation effect on patulin. Therefore, we hypothesized that strain BA-16-8 could reduce the amount of toxin accumulation by suppressing *P. expansum* from producing PAT [1,2].

To verify this hypothesis, the effect of fengycin on the mRNA expression level of *P. expansum* patulin synthesis gene (6-MSAS, IDH) was investigated by fluorescence q-PCR at the molecular level. As was shown in the results, the expression level of 6-MSAS decreased significantly under 50 μg/L fengycin, which was 0.11 times that of the control group. While the expression level of IDH decreased slightly, and the difference was not significant, it was 0.93 times that of the control group. Based on the above results, fengycin has been found to have a negative regulatory effect on the synthesis of patulin.

6-methylsalicylic acid synthase (6-MSAS) were synthesized from 81 molecules of acetyl-Coenzyme A (acetyl-coA) and three molecules of malonyl-coA, which catalyzed the first reaction of the penicillin biosynthetic pathway [24]. Malonyl-coA also played an important role in the extension stage of fatty acid biosynthesis and the synthesis of polyketones. It was necessary for the synthesis of fatty acids by acetyl-CoA. Fengycin, a lipopeptide antibiotic, had both peptide rings and fatty acid chains. Therefore, there exists a competition of malonyl-CoA between patulin and fengycin in their synthesis process [25]. As a result, fengycin may inhibit patulin synthesis by inhibiting gene expression of 6-MSAS in *P. expansum* mycelium [26]. The experimental results also confirmed that the addition of fengycin could down-regulate the expression of 6-MSAS. However, it did not significantly inhibit the expression of IDH, which was another key gene in the patulin synthesis pathway.

Understanding the relationship between bacterial antibacterial lipopeptide fengycin and mycotoxin synthesis will help find more effective ways to inhibit the production of fungi and toxins [27,28,29,30].

## 5 Conclusion

In this study, the effect of fengycin on the expression level of *P. expansum,* PAT synthetic gene (6-MSAS, ITH) mRNA at the molecular level was investigated by fluorescence q-PCR method. The study showed that it was 50 μg/L under the action of fengycin, the expression level of gene 6-MSAS decreased significantly, which was 0.11 times that of the control group. In relatively comparison, the expression level of IDH decreased slightly, but the difference was not significant, which was 0.93 times in the control group. Based on the above results, it is indicated that fengycin has a negative regulatory effect on the synthesis of PAT. 6-MSA (PatK) catalyzes the first reaction of the PAT biosynthetic pathway, which is the synthesis of 6-MSA from 81 molecules of acetyl-CoA and 3 molecules of malonyl-CoA. Malonyl-CoA also plays an important role in the extension stage of fatty acid biosynthesis and polyketide synthesis. It is necessary for acetyl-CoA to synthesize fatty acids. As a lipopeptide antibiotic, fengycin has a peptide ring and a fatty acid chain. Therefore, PAT and fengycin compete with malonyl-CoA during the synthesis process. Moreover, fengycin may inhibit PAT’s synthesis by inhibiting 6-MSAS gene expression. The experimental results also confirmed that the addition of fengycin did down-regulate the expression of 6-MSAS, but it had no obvious inhibitory effect on another key gene IDH in the PAT synthesis pathway. The relationship between the bacterial antimicrobial lipopeptide fengycin and mycotoxin synthesis was studied which will help to find more effective ways to inhibit the production of fungi and toxins. This study will provide a theoretical basis for biological control of postharvest diseases of apples produced by *P. expansum*.
